# Complex Inhibitor Protection of Some Steels in Hydrochloric Acid Solutions by 1,2,4-Triazole Derivatives

**DOI:** 10.3390/ma18020464

**Published:** 2025-01-20

**Authors:** Yaroslav G. Avdeev, Tatyana A. Nenasheva, Andrey Y. Luchkin, Andrey I. Marshakov, Yurii I. Kuznetsov

**Affiliations:** A.N. Frumkin Institute of Physical Chemistry and Electrochemistry, Russian Academy of Sciences, Moscow 119071, Russia; avdeevavdeev@mail.ru (Y.G.A.); tnenasheva@inbox.ru (T.A.N.); andreyluchkin23@gmail.com (A.Y.L.); yukuzn@gmail.com (Y.I.K.)

**Keywords:** corrosion, kinetics of electrode reactions, steel, hydrochloric acid, hydrogen absorption by steel, corrosion inhibitors

## Abstract

The behavior of low-carbon steels (LCSs), a high-strength steel and a nickel–chromium alloy in HCl solutions in the presence of N-containing organic substances has been studied. N-containing organic substances that comprise 1,2,4-triazole in their structure (substance I and substance II) provide comprehensive protection of various steel grades from corrosion and hydrogen absorption by the metal bulk in HCl solutions under both isobaric and isochoric conditions. All the compounds studied reduce, to varying degrees, the concentration of hydrogen adsorbed and absorbed by steel in HCl solutions. The most promising way to expand the scope of application is to use substance I in HCl solutions for protecting steels from high-temperature corrosion as a mixture with hexamethylenetetramine (HMT). In 2 M HCl (100 °C) under isochoric conditions, a mixture of compound I and HMT exhibited an excellent result: the degree of protection of LCS against corrosion was 99.5%. Substance I and its mixture with HMT protect steels not only in pure HCl solutions, but also in environments contaminated with hydrogen sulfide, which is especially important for the acid stimulation of wells in oil production.

## 1. Introduction

A unique feature of HCl solutions, which determines their wide application in the process cycles of modern enterprises, is their ability to quickly destroy many water-insoluble solid mineral phases consisting of metal oxides, metal hydroxides and salts [1,2,3,4,5,6,7]. It is of importance that the products of reactions of such mineral phases with hydrochloric acid are soluble in water, which contributes to their efficient degradation. Aqueous solutions of HCl are used to remove thermal scale from products made of iron-based alloys. They are employed to clean the internal surfaces of steel process equipment from rust and other mineral deposits, including scale. An important area of application of HCl solutions is the stimulation of hydrocarbon production by acid treatment of oil- and gas-bearing formations that are carbonate rocks. 

In these process operations, the extremely undesirable corrosive action of HCl solutions on steel products and equipment occurs. The corrosive action of acid solutions on steel structures can be reduced by adding metal corrosion inhibitors (MCIs). Various groups of compounds have been studied as MCIs of steels in acid solutions. They are presented in reviews [8,9,10,11] and in research articles [12,13,14,15,16,17,18]. 

The MCIs used in today’s production for preventing steel corrosion in highly aggressive environments should suppress the destruction of these materials that occurs as electrochemical reactions of iron with a corrosive environment and prevent the penetration of hydrogen released as a result of the cathodic reaction into the bulk of the metallic material [19,20,21,22,23,24]. For many iron-based alloys, bulk sorption of hydrogen can cause hydrogen embrittlement of the metals that changes their mechanical properties. This effect is often unacceptable in industrial operation. The MCIs that meet the requirements of modern production should provide comprehensive protection of metal structures and products, effectively prevent their reactions with the acid and suppress the absorption of the resulting hydrogen by the metals. The action of MCIs on hydrogen absorption by steels is indirectly assessed by a decrease in its penetration into the bulk of a metal material [25,26,27,28,29,30,31,32,33,34,35,36,37,38,39]. More objective information on the influence of MCIs on the bulk sorption of hydrogen by steels can be gained by direct measurement of its content in the metal bulk by various methods, including vacuum and electrochemical extraction. The simultaneous control of the mechanical characteristics of steels is an important element of such studies [40,41,42].

Triazole derivatives constitute a promising group of MCIs for iron-based alloys in HCl solutions that has been actively studied in recent years. The published works (Table 1) provide data on the protection of iron-based alloys by these compounds but do not report on the possibility of preventing the absorption of hydrogen released by them, which does not allow us to conclude that they are capable of providing comprehensive protection of metals.

**Table 1:** Protection of iron-based alloys in HCl solutions by triazole derivatives.

No.	MCI	Corrosive Environment	Iron-Based Alloys	Z, %	Ref.
1	(3-Bromo-4-fluoro-benzylidene)-1,2,4-triazol-4-yl-amine; (4-trifluoromethyl-benzylidene)-1,2,4-triazol-4-yl-amine and (2-fluoro-4-nitro-benzylidene)-1,2,4-triazol-4-yl-amine	0.5 M HCl (30–60 °C), 3.2 mM MCI	Mild steel (MS)	72.5–90.3	[43]
2	1-*p*-Tolyl-1H-1,2,3-triazol-4-yl) methanol	1 M HCl (25 °C), 1 mM MCI	MS	81	[44]
3	5-Hexylsulfanyl-1,2,4-triazole	1 M HCl (65 °C), 1 mM MCI	Carbon steel (CS)	89	[45]
4	5-((Furan-2-yl)methyleneamino)-2H-1,2,4-triazole-3-thiol and 5-((thiophen-2-yl)methyleneamino)-2H-1,2,4-triazole-3-thiol	1 M HCl (25 °C), 5 mM MCI	MS	86 and 90	[46]
5	4-Amino-1-((8-hydroxyquinolin-5-yl)methyl)-1,2,3-triazole-5-carboxylate	1 M HCl (25 °C), 1 mM MCI	CS	85	[47]
6	3,5-Bis(3-aminophenyl)-4-amino-1,2,4-triazole; 3,5-bis(4-aminophenyl)-4-amino-1,2,4-triazole; 3,5-bis(4-tolyl)-4-amino-1,2,4-triazole; 3,5-bis(4-methoxyphenyl)-4-amino-1,2,4-triazole and 3,5-bis(3,4-methoxyphenyl)-4-amino-1,2,4-triazole	1 M HCl (35 °C), 0.1 mM MCI	MS	89.9–98.5	[48]
7	5-Octylsulfanyl-1,2,4-triazole and 5-decylsulfanyl-1,2,4-triazole	1 M HCl (30 °C), 1 mM MCI	MS	93 and 92	[49]
8	3-(4-Ethyl-5-mercapto-1,2,4-triazol-3-yl)-1-phenylpropanone	1 M HCl (30 °C), 1000 ppm MCI	MS	97	[50]
9	1-Benzyl-4-phenyl-1H-1,2,3-triazole	1 M HCl (25 °C), 2.13 mM MCI	MS	81.4	[51]
10	2-(4-Phenyl-1H-1,2,3-triazol-1-yl) acetate and 2-(4-phenyl-1H-1,2,3-triazol-1-yl) acetohydrazide	1 M HCl (25 °C), 1 mM MCI	MS	95.3 and 95.0	[52]

Our study [53] shows that by using tribenzyl-1H-1,2,4-triazol-3-ammonium chloride and trioctyl-1H-1,2,4-triazol-3-ammonium bromide as MCIs in H_2_SO_4_ solutions, it is possible to provide comprehensive protection of various iron-based alloys.

We have shown [42] that the mechanism of action of 1,2,4-triazole derivatives in the protection of steels in acid solutions is based on the formation of a protective layer of inhibitor molecules on the metal surface. In these protective layers, the inhibitor molecules directly adjacent to the metal surface are bound to them by chemical bonds. Within the protective layer, the triazole molecules bind to the Fe(II) cations formed in the corrosion process to form an inorganic polymer complex. The polymer complex that forms the protective layer is basically composed of 1,2,4-triazole derivatives linked through the nitrogen atoms of the triazole ring to Fe(II) cations. The triazole ring acts as a bidentate ligand, which determines the polymeric structure of the complex.

The most effective mechanism for protecting steels in mineral acid solutions, including hot environments (with temperatures (*t*) up to 100 °C) that are important under industrial conditions, involves the formation of protective layers where the inhibitor molecules are bound to each other and to the metal surface by chemical interaction [42]. It is precisely such layers that 1,2,4-triazole derivatives are capable of forming on the metal surface. We made an assumption that this mechanism of action of 1,2,4-triazole derivatives would provide effective protection of various iron-based alloys from corrosion in HCl solutions in a wide temperature range. We also assumed that protective layers of 1,2,4-triazole derivatives formed on the metal surface would prevent the absorption of hydrogen released by the cathodic reaction during the corrosion of iron-based alloys. To confirm these assumptions, a comparative study of 1,2,4-triazole derivatives and structurally similar corrosion inhibitors that lack a triazole cycle in their structure was carried out.

It appears expedient to study 1,2,4-triazole derivatives (tribenzyl-1H-1,2,4-triazol-3-ammonium chloride and trioctyl-1H-1,2,4-triazol-3-ammonium bromide) as complex MCIs in HCl solutions. It is important to demonstrate the possibility of their use in HCl solutions in a wide temperature range of 25–100 °C, which is important for the industrial operation of these environments. When HCl solutions are used at oil and gas enterprises, they become contaminated with hydrogen sulfide. Contamination of hydrochloric acid solutions with hydrogen sulfide occurs because it dissolves mineral deposits containing metal sulfides. Contamination of acid solutions with dissolved hydrogen sulfide often leads to a decrease in the effectiveness of inhibitor protection of metals in these solutions. MCIs providing complex protection should retain their properties in these environments. It is necessary to consider whether it is possible to improve the protective action of the compounds studied by creating mixed corrosion inhibitors for metals on their basis. We studied hexamethylenetetramine (HMT) as an additive possessing such properties. HMT is known as an effective additive that improves the protective action of metal corrosion inhibitors in hydrochloric acid solutions [54].

In manufacturing plants, items and equipment made of iron-based alloys can contact hydrochloric acid solutions both under isobaric conditions (etching of metals in open containers with acid solutions) and under isochoric conditions (flushing of closed pipeline systems or hydrochloric acid stimulation of oil wells). It is important to understand how the corrosion inhibitors in question will protect iron-based alloys in HCl solutions under isobaric and isochoric conditions. In this regard, the corrosion of iron-based alloys in HCl solutions was studied in open vessels under atmospheric pressure and in an autoclave unit under constant volume conditions.

## 2. Research Objects and Methods

### 2.1. Materials

Hydrochloric acid solutions with various concentrations were examined as the corrosive environments. In some cases, HCl solutions containing dissolved H_2_S were used. The effect of MCIs on the corrosion process was studied for various iron-based alloys: low-carbon steels (LCSs), a high-strength steel (HSS) and a nickel–chromium (Ni-Cr) alloy. The chemical composition of the metal materials is presented in Table 2.

**Figure 1:**
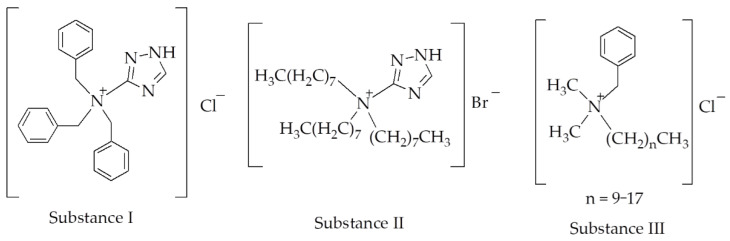
Chemical formulas of the MCIs studied.

Two 1,2,4-triazole derivatives were studied as MCIs and compounds that suppress hydrogen absorption by iron-based alloys in HCl solutions, tribenzyl-1H-1,2,4-triazol-3-ammonium chloride (substance I) and trioctyl-1H-1,2,4-triazol-3-ammonium bromide (substance II), as well as one that does not comprise a 1,2,4-triazole ring structure, i.e., substance III (a mixture of alkylbenzyldimethylammonium chlorides) (Figure 1).

The solutions were prepared using distilled water, concentrated HCl solution, HMT and gaseous H_2_S obtained by the reaction of sodium sulfide with 20% H_2_SO_4_. The solutions were prepared from “chemically pure”-grade reagents. We studied 2 M HCl solutions containing 1, 5 and 15 mM H_2_S. The content of hydrogen sulfide in HCl solutions was measured by the iodometric method. H_2_S reacted with excess I_2_ + KI solution of a known concentration. The content of unreacted I_2_ in the mixture was determined by titration with a Na_2_S_2_O_3_ solution in the presence of starch solution as the indicator [55]. Voltammetric studies were carried out in HCl solutions de-aerated with argon gas.

### 2.2. Methods

#### 2.2.1. Quantitative Indicators of Corrosion of Metal Alloys at Constant Pressure

The progress of steel corrosion over time was judged by the mass loss of metal samples upon exposure to a corrosive environment. The experiments were carried out in open vessels under isobaric conditions at atmospheric pressure. The corrosion rate (CR) was calculated using data on the mass loss of the samples:

(1) $$\mathrm{CR}=\frac{∆m}{S\tau }$$

where Δm is the mass loss of a metal coupon upon contact with the acid solution; S is the area of a metal coupon surface that contacts the acid; and τ is the duration of contact of the metal coupon with the acid solution. 

The effect of substances and their mixtures with HMT on the corrosion of alloys was characterized by the following degree of protection:

(2) $$Z=\frac{{\mathrm{CR}}_{0}-{\mathrm{CR}}_{\mathrm{MCI}}}{{\mathrm{CR}}_{0}}100\%$$

where ${\mathrm{CR}}_{\mathrm{MCI}}$ and ${\mathrm{CR}}_{0}$ are the corrosion rates in a solution with and without an MCI, respectively, in g/(m^2^·h) [54].

#### 2.2.2. Determination of the CR Based on Autoclave Corrosion Test Data

The corrosion rate of iron-based alloys in hydrochloric acid solutions under constant volume conditions was measured using an automatically controlled autoclave system. The CR of alloys in autoclave corrosion tests was determined from Equation (1). Equation (2) was used to give a quantitative characteristic of the influence of the studied substances and their mixtures with HMT on the corrosion of alloys.

#### 2.2.3. Vacuum Extraction Method

The content of absorbed hydrogen in iron-based alloys was determined by measuring the desorption of hydrogen upon heating (500 °C) in a quartz setup equipped with a McLeod mercury manometer, inside which a vacuum was created initially. The amount of hydrogen in the alloys (mL/0.100 kg of metal) was calculated using the following formula:

(3) $$V=\frac{\mathrm{Gp}}{m}$$

where G is a parameter determined by the geometry of the vacuum setup; p is the pressure of desorbed H_2_; and m is the mass of the metal coupon [54].

#### 2.2.4. Mechanical Properties of Alloys

The mechanical properties of iron-based alloys were determined by their tolerance to cyclic bending of metal coupons. The plasticity of the material (π, %) was characterized by the number of bends of tape coupons until failure:

(4) $$\pi =\frac{\beta }{\beta_{0}}100\%$$

where β and β_0_ denote the number of cyclic bends withstood by alloy coupons after exposure to the acid solution and by original coupons without such exposure, respectively [54].

#### 2.2.5. Voltammetry

To study the electrochemical behavior of iron-based alloys of different compositions both in the background HCl solution and in such solutions containing corrosion inhibitors, a potentiostat-galvanostat EL 02.061 (“ELNITEKS”, Moscow, Russia) was used. Cathodic and anodic polarization curves were obtained in potentiodynamic mode (potential scan rate 0.5 mV/s). The voltammetric curves were recorded after keeping the metal electrode at the corrosion potential (E_cor_) for 30 min. First, the anodic voltammetric curve was obtained by shifting the metal potential (E) from E_cor_ to 0 V (HSS) or 0.05 V (Ni-Cr alloy). Then, the cathodic voltammetric curve was recorded by shifting the metal potential from E_cor_ to −0.4 V (HSS and Ni-Cr alloy). The studies were carried out under temperature-controlled conditions (60 °C). A glass cell with divided spaces for the auxiliary and reference electrodes (smooth platinum) was used. A platinum electrode was used as the auxiliary electrode, and a saturated silver chloride electrode was used as the reference electrode.

#### 2.2.6. Permeation Test

A two-compartment glass cell was used to study the effect of various additives on the amount of hydrogen absorbed by the metal in HCl solutions, using the method developed by Devanathan and Stachurski [56,57]. The working electrode was a membrane made of a low-carbon iron alloy (0.1 mm thick, 4.25 cm^2^ area). Preparation of the working electrode and the experimental procedure are described in detail elsewhere [40]. 

#### 2.2.7. IPZ Analysis Method

Iyer et al. proposed the Iyer–Pickering–Zawenzaden (IPZ) analysis to quantitatively measure the properties of hydrogen on a metal surface, mainly the surface coverage and the related kinetic parameters [58,59]. The calculations were based on the experimental data obtained by the Permeation Test (2.2.6). The IPZ analysis, which was used to calculate the kinetic constants of H^+^ ion discharge (k_1,i_), the molization of hydrogen atoms (k_r_), exchange constants (k), surface hydrogen coverage ($\theta_{H}$) and subsurface hydrogen concentration ($C_{H}^{s}$) in acids containing organic corrosion inhibitors, is described in detail elsewhere [40].

#### 2.2.8. Determination of the Resistance of Alloys to Hydrogen Absorption

The effectiveness of the inhibitor was judged by the hydrogen content in the volume of the alloys studied (V) (Section 2.2.3),

(5) $$Z_{H}^{V}=\frac{V-V_{\mathrm{MCI}}}{V}100\%$$

and the molar concentration of H atoms under the iron-based alloy surface ($C_{H}^{s}$) (Section 2.2.7)

(6) $$Z_{H}^{s}=\frac{C_{H}^{s}-C_{H,\;\mathrm{MCI}}^{s}}{C_{H}^{s}}100\%$$

where V and $V_{\mathrm{MCI}}$ indicate the hydrogen content in a iron-based alloy after exposure in the blank environment and in an environment containing an MCI, respectively; $C_{H}^{s}$ and $C_{H,\;\mathrm{MCI}}^{s}$ are the subsurface concentrations of hydrogen in an iron-based alloy after exposure in the same respective environments.

#### 2.2.9. AFM Method

Surface micrographs were taken using the built-in camera of a SolverNext II atomic force microscope (NovaPhotonix LLC, Moscow, Russia). A silicon probe (resonance frequency 73 kHz, elasticity coefficient 4.5 N/m) was used. The cantilever potential was 4.72 V.

The electron work function characterized by the measured surface potential was determined from the equation

(7) $$V_{\mathrm{pp}}=\frac{W_{c}-W_{s}}{e}$$

where $W_{c}$ is the electron work function of the probe material; $W_{s}$ is the electron work function of the metal coupon; and *e* is the elementary electric charge.

## 3. Results and Discussion

### 3.1. Corrosion of LCS Under Constant Pressure

The corrosion of St3 steel in 2 M HCl under isobaric conditions accelerates with increasing temperature (Table 3). The best protection of the metal in a hot solution of 2 M HCl (60–100 °C) is observed in the presence of 5 mM of substance I as the additive. Substance III shows the worst result under the same conditions. At t = 100 °C in the presence of 5 mM MCIs, the values of CR are quite high. Therefore, a higher concentration of the MCIs was investigated. At t = 100 °C, satisfactory protection of St3 steel is provided only by the addition of 10 mM of substance I, which results in k = 16 g/(m^2^·h).

The corrosion of St3 steel in HCl solutions (t = 60 °C) of various concentrations accelerates with increasing hydrogen chloride content (Table 4). The high efficiency of substances I and II, which are 1,2,4-triazole derivatives, in slowing down metal corrosion in HCl solutions containing 1-6 M hydrogen chloride was demonstrated. In the presence of substance III, which is not a triazole derivative, the CR values are higher.

### 3.2. Corrosion of Alloys Under Isochoric Conditions

Metal process equipment is cleaned with acid solutions from mineral contamination both in open and closed systems. Accordingly, the metal corrosion process was implemented under isobaric and isochoric conditions. The MCIs we studied inhibit LCS corrosion in HCl solutions under constant pressure. It is necessary to understand what protective effects the substances we are considering will provide in case of metal corrosion in closed systems, including the processes occurring in heated systems. In the case of acid solutions, the processes corresponding to high-temperature corrosion (80–100 °C) are of particular interest. In practice, such systems include the internal volumes of pipelines cleaned with acidic formulations, or oil wells subjected to the acid stimulation procedure. In laboratory practice, autoclaves are used to create conditions that meet these procedures. Under such conditions, the CR of LCS in HCl solutions is high (Table 5). At temperatures up to 100 °C and for different testing times, the CR ranges within 1.4–1.6 kg/(m^2^·h). 

We selected substance I, which is the most efficient compound of those studied earlier, for testing in this study. Data for substance III, which shows the worst result, are also provided for a more detailed understanding. It is evident that at t = 80 °C, substance I (10–20 mM) is efficient in slowing down corrosion. However, at t up to 100 °C, the corrosion of LCS in the HCl solution containing substance I increases sharply. A more interesting result is provided by a mixture of 10 mM of substance I + 10 mM HMT. At t up to 100 °C in a long-term corrosion test (2 h), the values of CR are ≤7.1 g/(m^2^·h), which is an extremely important result. HMT alone (20 mM) is a poor corrosion inhibitor. It should be noted that under the testing conditions, 20 mM of substance I and, even more so, 10 mM of substance I + 10 mM HMT reduce the corrosion of St20 steel significantly better than 20 mM of substance III.

The acid solutions circulating in process equipment dissolve metal sulfides and become contaminated with dissolved H_2_S. It is important to understand how the presence of dissolved H_2_S in the cleaning acid will affect the protective action of the MCIs added to it. The presence of hydrogen sulfide in the background HCl solution increases the steel corrosion rate (Table 6). In inhibited 2.0 M HCl solutions, hydrogen sulfide slightly reduces the protective effect of substance I and its mixture with HMT. However, even under these conditions, it is smaller than in the background solution by a factor of no less than 85. In general, despite some weakening of the protective effect of substance I and its mixture with HMT, they retain high efficiency in inhibiting LCS destruction in the presence of such an aggressive component as hydrogen sulfide.

### 3.3. Effect of MCIs on Hydrogen Absorption by Alloys

The nitrogen-containing organic compounds containing a 1,2,4-triazole cycle significantly reduce the corrosion rate of LCSs in HCl solutions under isobaric and isochoric conditions. It is necessary to obtain information on the hindrance of hydrogen absorption by corroding iron-based alloys with substance I and substance II. HHS is often subject to hydrogen embrittlement, which determined the choice of 70S2KhA steel for use in the tests. In parallel, LCS of grade 08PS and the Cr-Ni alloy were tested.

In 2 M HCl (60 °C), the addition of substances I and II protects HSS, decreasing the CR 20- and 21-fold, respectively (Table 7). At the same time, the amount of hydrogen contained in steel coupons is greatly reduced in comparison with metal coupons after exposure to a solution without the test substances. The degrees of inhibition of hydrogen absorption by 70S2KhA steel are 88 and 90%, respectively. Substance III is inferior to substances I and II in its ability to protect steel, reduce hydrogen absorption and preserve plasticity.

In 2 M HCl (25 °C and 60 °C), triazole derivatives slow down the corrosion of 08PS steel better than substance III (Table 8). Hydrogen absorption by LCS virtually does not occur in the environment without the test substances. Some substances of organic and inorganic nature, while being efficient corrosion inhibitors, do not have a significant effect on hydrogen absorption by the metal, or sometimes even accelerate it. All the organic additives we studied do not promote hydrogen absorption by 08PS steel. In the presence of substance I and substance II, the CR of LCS is the smallest.

The production equipment of various modern facilities can be partially or completely made of Ni-Cr alloys. Such alloys may be prone to corrosion in HCl solutions (Table 9). Substances I and II in a hot solution of 2 M HCl significantly suppress the corrosion of stainless steel 1Kh18N9T and completely prevent hydrogen absorption by it. Substance III slows down the corrosion of steel 1Kh18N9T inefficiently and stimulates hydrogen absorption by this steel.

In 2 M HCl + 15 mM H_2_S (25–60 °C), the addition of 5 mM of substance I slows down the corrosion of 1Kh18N9T steel efficiently and completely prevents hydrogen absorption by it at various durations of the corrosion tests (Table 10). Hydrogen absorption by the metal is only observed in long-term corrosion tests (2 h) in 2 M HCl + 15 mM H_2_S (60 °C). The use of the mixture of 1 mM of substance I + 4 mM HMT prevents hydrogen absorption by steel completely at any of the selected test durations.

Complex protection of iron-based alloys of various grades can be achieved by using triazole derivatives as MCIs. Such MCIs suppress the interaction of iron-based alloys with acid solutions and reduce the amount of hydrogen dissolved in the metal material. In the case of substance I or its formulation with HMT, this effect is preserved even in HCl solutions containing hydrogen sulfide. This is an important practical result encouraging a more detailed study of such substances as MCIs in acid environments.

### 3.4. The Influence of MCIs on the Mechanical Properties of HSS

In many cases, the dissolution of hydrogen in iron-based alloys changes their mechanical properties significantly. An increase in the content of atomic hydrogen in the bulk of HSS of grade 70S2KhA results in a complete loss of plasticity after exposure in the background HCl solution (Table 7). The addition of substances I and II to this environment leads to complete preservation of HSS plasticity. This result correlates with the low content of dissolved hydrogen in HSS. Substance III does not prevent the loss of HSS plasticity due to the relatively high content of dissolved hydrogen in the material.

### 3.5. Electrode Reactions of Alloys

**Figure 2:**
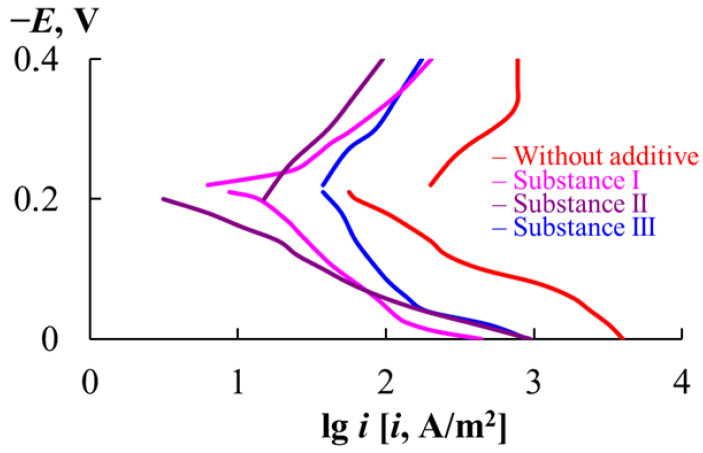
Voltammetric curves of HSS in 2 M HCl (60 °C).

Important information about the specifics of metal corrosion in various aggressive electrolytes containing MCIs can be obtained using the voltammetric method. In the HSS/2 M HCl corrosion system under consideration, material destruction occurs in the active dissolution region (Table 11, Figure 2). The slopes of the voltammetric curves approximately amount to 120 and 210 mV for the anodic and cathodic reactions, respectively.

The corrosion potential (E_cor_) of HSS in the presence of substances I, II and III is close to the value observed in the solution without MCIs. Like in the background environment, the slopes of the anodic and cathodic voltammetric curves in the presence of substances I, II and III are high (b_a_ = 95–270 mV and b_c_ = 180–270 mV). The degree of protection of the MCIs for the anodic and cathodic reactions on HSS decreases in the series substance II > substance I > substance III, which is consistent with the results on the corrosion of HSS calculated from the mass loss of the samples (Table 7).

**Figure 3:**
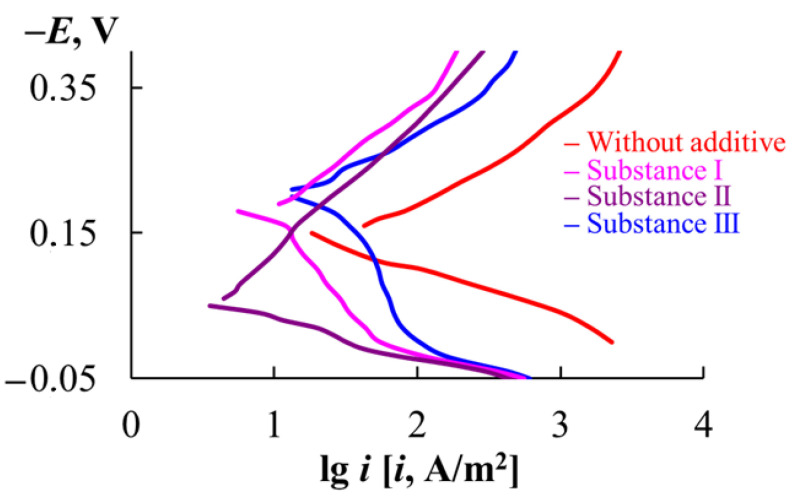
Voltammetric curves of the Ni-Cr alloy in 2 M HCl (60 °C).

In 2 M HCl (60 °C), the E_cor_ of the Ni-Cr alloy is in the potential region of active dissolution (Table 11, Figure 3). Substances I and III shift the E_cor_ of the Ni-Cr alloy to more negative values compared to the environment without MCIs, which is typical of substances that predominantly slow down the cathodic process of a metal. In contrast, substance II shifts the E_cor_ to more positive values compared to the background environment. This indicates that substance II predominantly inhibits the anodic reaction of the metal. The degree of protection of the organic substances studied on the anodic reaction decreases in the series substance II > substance I > substance III, and for the cathodic reaction of steel it decreases in the series substance I > substance II > substance III. The triazole derivatives provide the greatest effect in inhibiting the electrode reactions. The overall result of such an effect of triazole-containing substances on the electrode processes of the Ni-Cr alloy is that they inhibit the general corrosion of the metal more significantly than substance III.

The significant hindrance of the electrode reactions of the HSS and Ni-Cr alloy by substances I and II results from the peculiarities of the chemical structure of these compounds. The presence of a 1,2,4-triazole fragment in the structure of these MCIs ensures their strongest bond with the surface atoms of iron-based alloys, as we have shown previously [41,42] using physicochemical methods (EIS and XPS spectroscopy).

### 3.6. Kinetics of Reactions of Hydrogen Evolution and Penetration into the LCS

The effect of metal corrosion inhibitors on cathodic hydrogen evolution on low-carbon steel in the HCl solution was studied at their concentration of 5 mM. The experiments were carried out in a two-compartment glass cell at a temperature of 25 °C.

**Figure 4:**
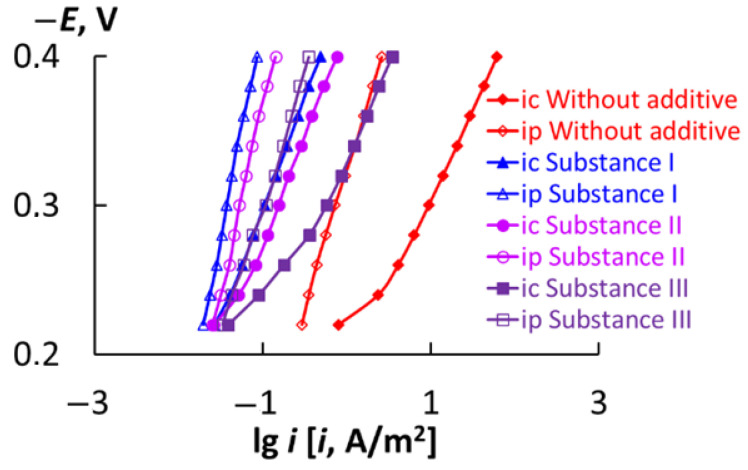
Cathodic current on St3 and the current of hydrogen penetration into the LCS vs. potential in 2 M HCl solutions.

The experimental plots of the hydrogen discharge current and H penetration into the metal vs. potential in the background HCl solution without and with the corrosion inhibitors are displayed in Figure 4. In the presence of all the substances studied, the rates of the cathodic reaction (i_c_) and the current density of hydrogen penetration into the metal (i_p_) slow down significantly. Substance I exhibits the greatest efficiency; if it is present in hydrochloric acid, the cathodic current density decreases 90-fold, while the rate of H penetration into the high-strength steel decreases 20-fold. A good result is also observed for substance II (the i_c_ value decreases by a factor of 50, and i_p_ by a factor of 15). Although substance III is less efficient than the other two compounds, it also slows down both the cathodic release of hydrogen (by a factor of 15) and hydrogen absorption by the metal (by a factor of 7).

To determine the concentration of H adsorbed and absorbed by the high-strength steel in solutions with various compositions, it is necessary to know the main constants of the reaction of cathodic hydrogen evolution on the metal in specific solutions. If the solution contains organic compounds, such as metal corrosion inhibitors (MCIs), the degree of coverage of the metal surface with an MCI itself ($\theta_{\mathrm{inh}}$) must be taken into account [40]. $\theta_{\mathrm{inh}}$ were determined from the ratio of the (i_c,0_) value in the background HCl solution to the (*i*_c,inh_) values in solutions containing various MCIs [60].

(8) $$\theta_{\mathrm{inh}}=\frac{i_{c,0}-i_{c,\mathrm{inh}}}{i_{c,0}}100\%$$

The calculated values of $\theta_{\mathrm{inh}}$ were 0.98, 0.99 and 0.95 for substances I, II and III, respectively. 

### 3.7. Calculation of the Main Kinetic Constants of Reactions of Cathodic Release and Penetration of Hydrogen into the Metal

The IPZ analysis method [40,58] allows for the calculation of the reaction constants of proton cathodic reduction based on data of cathodic curves and the values of the current of hydrogen diffusion into the LCS (Figure 4). The rate constants of hydrogen ion discharge (k_1,i_), the rate constants of the molization of hydrogen atoms (k_r_) and the exchange constants (k) were calculated both in the background 2M HCl solution and in solutions containing various MCIs (Table 12). All the compounds studied change these constants. The rates of H^+^ discharge and H atom molization decreases. The values of exchange constants increase.

Using the values of the constants (Table 12) and the equations presented in [40], the amount of hydrogen on the surface of the metal ($\theta_{H}$), the subsurface hydrogen concentration in the metal ($C_{H}^{s}.$) and the coefficient of inhibition of hydrogen absorption by the metal ($Z_{H}^{s}.$) were calculated. 

As follows from Table 12, all the compounds studied reduce the amounts of both hydrogen adsorbed on the metal surface ($\theta_{H}$) and hydrogen absorbed in the metal bulk ($C_{H}^{s}.$). The efficiency of the compounds studied varies, but the degree of protection of steel from hydrogen absorption by the metal reaches significant values: $Z_{H}^{v}.$ = 85–96.5%. 

### 3.8. Atomic Force Microscopy

Micrographs and topographic maps of the surface along with the surface roughness values and calculated work function values are presented in Table 13. 

Etching a steel sample in 2M HCl results in uniform dissolution of the metal surface. This conclusion can be made from a visual assessment of the sample and from the micrograph that shows minor amounts of sludge, while the original steel relief, including scratches, is preserved. At the same time, the *s* value remains virtually unchanged, reaching 3.95 nm. The calculated W_s_ value increases by 0.208 eV, probably due to the presence of sludge on the metal.

The presence of substances I, II and III in the HCl solution results in the emergence of pronounced, fairly large streaks and drops on the metal surface, which are clearly visible even in the micrographs. Analysis of AFM images shows a twofold increase in *s* and an increase in W_s_ for all the compounds studied. For steel samples exposed to inhibited HCl solutions, the W_s_ values differ significantly and can be arranged in a series of increasing W_s_: substance II ˂ substance I ˂ substance III. After etching the sample in 2 M HCl + 5 mM substance III, the sample surface is etched to the largest extent and individual cracks are even observed. Thus, based on atomic force spectroscopy data, it is difficult to draw a conclusion as to which of the organic compounds is more efficient in slowing down corrosion, since the values are very close to each other.

According to microscopy data, metal etching in a background acid solution and in an acid solution containing substance III does not change the surface roughness significantly. This is explained by the fact that hydrochloric acid solutions etch the surface fairly uniformly. It is somewhat unusual that after etching metal samples in acid solutions containing substances I and II, the surface roughness increases. At the same time, according to data on the rate of metal corrosion in such environments, the metal loss from its surface is minor, and metal etching should not occur. This effect is not the result of a change in the metal surface in the course of etching. It is due to the formation of a protective inhibitor layer consisting of a 1,2,4-triazole derivative on the steel surface. The protective layer is unevenly distributed over the metal surface, which is visible in the photographic images. As a result, the surface roughness measured by the device is higher than that for samples exposed to the background environment.

## 4. Conclusions

1. All the nitrogen-containing organic compounds studied exhibit a high protective effect for steels with various compositions against corrosion and hydrogen embrittlement in hydrochloric acid solutions (1–8 M) in the open atmosphere and at elevated pressure. Substance I, both alone and as a mixture with HMT, inhibits steel corrosion in HCl solutions. Organic compounds comprising a triazole ring (substances I and II) can efficiently protect the LCS, the HSS and the Ni-Cr alloy from corrosion and hydrogen absorption in hydrochloric acid solutions (1–8 M) in a wide temperature range (25–100 °C) under isobaric and isochoric conditions. In some cases, the degree of protection of steels by substance I is almost 99%. The possibility of creating mixed inhibitors for steel protection containing a triazole derivative and HMT has been shown.
2. Triazole derivatives (substances I and II) slow down the anodic and cathodic processes and penetration of hydrogen into the iron-based alloys in hydrochloric acid solutions.
3. Substance I shows high inhibitory efficiency in HCl solutions containing hydrogen sulfide.
4. Using the kinetic constants of the stages of hydrogen cathodic evolution and diffusion into the LCS, the amount of hydrogen adsorbed and absorbed by the metal in a hydrochloric acid solution containing the MCIs was determined. All the compounds studied efficiently inhibit the penetration of hydrogen into the LCS. The degree of LCS protection against hydrogen absorption reaches 96.5%.
5. Triazole derivatives are complex inhibitors of steel corrosion in hydrochloric acid solutions since, along with strong inhibition of metal corrosion, they prevent the absorption of hydrogen by the metals. They do not lose their protective effect at temperatures up to 100 °C and can provide protection in a wide range of acid concentrations (1–8 M). They manifest protective effects for various grades of steel. It is important that the protective effect of substance I is enhanced when it is combined with HMT. This opens up opportunities to create mixed corrosion inhibitors comprising it, which significantly reduces the consumption of the components being mixed.
6. The high efficiency of 1,2,4-triazole derivatives (substances I and II) in slowing down the corrosion of various steels is a result of their ability to form surface protective layers consisting of a polymer complex of a triazole derivative and Fe(II) cations. Substance III, which does not contain a triazole cycle in its structure, is significantly inferior in protection to substances I and II.

## Figures and Tables

**Table 2:** Chemical composition of the iron-based alloys.

Steel Grade	Chemical Elements, Mass (%)								
	C	Si	Mn	Ni	S	P	Cr	Cu	Ti
LCS									
St3	0.14–0.22	0.15–0.3	0.4–0.65	≤0.3	≤0.05	≤0.04	≤0.3	≤0.3	≤0.8
St20	0.17–0.24	0.17–0.37	0.35–0.65	≤0.25	≤0.04	≤0.04	≤0.25	≤0.25	-
08PS	0.05–0.11	0.05–0.11	0.35–0.65	≤0.3	≤0.04	≤0.04	≤0.1	≤0.3	-
HSS									
70S2KhA	0.65–0.75	1.4–1.7	0.4–0.6	≤0.25	≤0.03	≤0.03	0.2–0.4	≤0.2	-
Ni-Cr alloy									
1Kh18N9T	≤0.12	≤0.8	≤2.0	9–11	≤0.02	≤0.04	17–19	≤0.3	-

**Table 3:** CR of St3 steel in 2 M HCl under isobaric conditions.

Corrosion Parameters	MCI (5 mM)			
	Without MCI	Substance I	Substance II	Substance III
25 °C				
CR, g/(m^2^·h)	5.1	0.39	0.36	0.80
Z, %	-	92.4	92.9	84.3
60 °C				
CR, g/(m^2^·h)	50	0.68	1.6	2.9
Z, %	-	98.6	96.8	94.2
80 °C				
CR, g/(m^2^·h)	380	4.6	6.2	21
Z, %	-	98.8	98.4	94.5
100 °C				
CR, g/(m^2^·h)	1300	22	34	81
Z, %	-	98.3	97.4	93.8
CR, g/(m^2^·h)	1300	16 *	24 *	69 *
Z, %	-	98.8 *	98.2 *	94.7 *

* The concentration of the substances is 10 mM.

**Table 4:** CR of St3 steel in HCl solutions of various concentrations under isobaric conditions (60 °C).

Corrosion Parameters	HCl Content, M				
	1	2	4	6	8
Without MCI					
CR, g/(m^2^·h)	38	50	180	390	590
5 mM Substance I					
CR, g/(m^2^·h)	0.42	0.68	1.1	6.3	30
Z, %	98.9	98.6	99.4	98.4	94.9
5 mM Substance II					
CR, g/(m^2^·h)	0.63	1.6	3.3	9.0	39
Z, %	98.3	96.8	98.2	97.7	93.4
5 mM Substance III					
CR, g/(m^2^·h)	6.3	2.9	8.4	32	130
Z, %	84.2	94.2	95.3	91.8	78.0

**Table 5:** CR of St20 steel in 2 M HCl under isochoric conditions.

Corrosion Parameters	Without MCI	Substance I		Substance I + HMT	HMT	Substance III
		10 mM	20 mM	10 mM + 10 mM	20 mM	20 mM
Exposure time of metal samples in acid solution: 0.5 h; 80 °C						
CR, g/(m^2^·h)	400	3.7	3.3	2.9	38	23
Z, %	-	99.1	99.2	99.3	90.5	94.3
Exposure time of metal samples in acid solution: 1.0 h; 80 °C						
CR, g/(m^2^·h)	400	3.3	3.2	2.4	41	25
Z, %	-	99.2	99.2	99.4	89.8	93.8
Exposure time of metal samples in acid solution: 2.0 h; 80 °C						
CR, g/(m^2^·h)	370	3.0	2.5	1.7	45	28
Z, %	-	99.2	99.3	99.5	87.8	92.4
Exposure time of metal samples in acid solution: 0.5 h; 100 °C						
CR, g/(m^2^·h)	1600	28	27	11	200	120
Z, %	-	98.3	98.3	99.3	87.5	92.5
Exposure time of metal samples in acid solution: 1.0 h; 100 °C						
CR, g/(m^2^·h)	1500	27	24	8.1	240	130
Z, %	-	98.2	98.4	99.5	84.0	91.3
Exposure time of metal samples in acid solution: 2.0 h; 100 °C						
CR, g/(m^2^·h)	1400	27	23	7.1	340	140
Z, %	-	98.1	98.4	99.5	75.7	90.0

**Table 6:** Corrosion of St20 steel in HCl + H_2_S under isochoric conditions. Exposure time of metal samples in acid solution: 2 h (80 °C).

Corrosion Parameters	Without MCI	Substance I	Substance I + HMT
		10 mM	10 mM + 10 mM
2 M HCl			
CR, g/(m^2^·h)	370	3.0	1.7
Z, %	-	99.2	99.5
2 M HCl + 1 mM H_2_S			
CR, g/(m^2^·h)	380	4.3	3.6
Z, %	-	98.9	99.1
2 M HCl + 5 mM H_2_S			
CR, g/(m^2^·h)	450	5.3	4.0
Z, %	-	98.8	99.1

**Table 7:** Corrosion of HSS in 2 M HCl. Time of exposure of metal samples in acid solution: 2 h (25 °C).

Corrosion Parameters	MCI (5 mM)			
	Without MCI	Substance I	Substance II	Substance III
CR, g/(m^2^·h)	12	0.59	0.58	6.4
Z, %	-	95.1	95.1	46.7
Volume of hydrogen absorbed in steel *, mL/0.100 kg of metal	4.62	0.56	0.46	1.72
Inhibition of hydrogen absorption, %	-	88	90	63
Ductility of metal, %	-**	100	100	-

* The data are corrected for metallurgical hydrogen, which is 0.34 mL/0.100 kg of metal. ** Complete loss of plasticity of metal coupons.

**Table 8:** Corrosion of 08PS steel in 2 M HCl. Time of exposure of metal samples in acid solution: 2.0 h.

Corrosion Parameters	MCI (5 mM)			
	Without MCI	Substance I	Substance II	Substance III
Temperature of the corrosive environment—25 °C				
CR, g/(m^2^·h)	2.3	0.13	0.16	0.22
Z, %	-	94.3	93.0	90.4
Volume of hydrogen absorbed in steel *, mL/0.100 kg of metal	H_Me_ **	H_Me_	H_Me_	H_Me_
Inhibition of hydrogen absorption, %	-	100	100	100
Temperature of the corrosive environment—60 °C				
CR, g/(m^2^·h)	54	0.69	0.92	3.3
Z, %	-	98.7	98.3	93.9
Volume of hydrogen absorbed in steel *, mL/0.100 kg of metal	H_Me_	H_Me_	H_Me_	H_Me_
Inhibition of hydrogen absorption, %	-	100	100	100

* The data are corrected for metallurgical hydrogen, which is 0.57 mL/0.100 kg of metal. ** H_Me_—the concentration of hydrogen in metal coupons does not exceed the content of metallurgical hydrogen.

**Table 9:** Corrosion of the Ni-Cr alloy in 2 M HCl. Time of exposure of metal samples in acid solution: 2.0 h (60 °C).

Corrosion Parameters	MCI (5 mM)			
	Without MCI	Substance I	Substance II	Substance III
CR, g/(m^2^·h)	9.2	0.49	0.57	2.3
Z, %	-	94.7	93.8	75.0
Volume of hydrogen absorbed in steel *, mL/0.100 kg of metal	0.71	0	0	1.71
Inhibition of hydrogen absorption, %	-	100	100	−140

* The data are corrected for metallurgical hydrogen, which is 2.33 mL/0.100 kg of metal.

**Table 10:** Corrosion of Ni-Cr alloy in 2 M HCl + 15 mM H_2_S.

Parameters of Corrosion	Time of Exposure of Metal Samples in Acid Solution			
	15 min	30 min	1.0 h	2.0 h
2 M HCl + 15 mM H_2_S; 20 °C				
CR, g/(m^2^·h)	7.2	5.1	4.1	3.2
Volume of hydrogen absorbed in steel *, mL/0.100 kg of metal	5.1	7.2	15	17
2 M HCl + 15 mM H_2_S + 5 mM Substance I; 20 °C				
CR, g/(m^2^·h)	0.40	0.32	0.26	0.22
Z, %	94.4	93.7	93.7	93.1
Volume of hydrogen absorbed in steel, mL/0.100 kg of metal	H_Me_ **	H_Me_	H_Me_	H_Me_
2 M HCl + 15 mM H_2_S; 40 °C				
CR, g/(m^2^·h)	15	13	9.7	8.1
Volume of hydrogen absorbed in steel *, mL/0.100 kg of metal	7.0	15	22	24
2 M HCl + 15 mM H_2_S + 5 mM Substance I; 40 °C				
CR, g/(m^2^·h)	1.1	0.80	0.57	0.38
Z, %	92.7	93.8	94.1	95.3
Volume of hydrogen absorbed in steel, mL/0.100 kg of metal	H_Me_	H_Me_	H_Me_	H_Me_
2 M HCl + 15 mM H_2_S; 60 °C				
CR, g/(m^2^·h)	62	57	52	62
Volume of hydrogen absorbed in steel *, mL/0.100 kg of metal	12	14	15	17
2 M HCl + 15 mM H_2_S + 5 mM Substance I; 60 °C				
CR, g/(m^2^·h)	1.4	0.85	0.66	0.61
Z, %	97.7	98.5	98.7	99.0
Volume of hydrogen absorbed in steel, mL/0.100 kg of metal	H_Me_	H_Me_	H_Me_	2.5
2 M HCl + 15 mM H_2_S + 1 mM Substance I + 4 mM HMT; 60 °C				
CR, g/(m^2^·h)	1.3	1.0	0.73	0.51
Z, %	97.9	98.2	98.6	99.2
Volume of hydrogen absorbed in steel, mL/0.100 kg of metal	H_Me_	H_Me_	H_Me_	H_Me_

* The data are corrected for metallurgical hydrogen, which is 2.33 mL/0.100 kg of metal. ** H_Me_—the concentration of hydrogen in metal coupons does not exceed the content of metallurgical hydrogen.

**Table 11:** Parameters of electrode reactions on HSS and Ni-Cr alloy in 2 M HCl (60 °C).

MCI (5 mM)	E_cor_, V	b_a_, V	b_c_, V	i_a_ *, A/m^2^	i_c_, A/m^2^	Z_a_, %	Z_c_, %
HSS							
Without MCI	−0.215	0.120	0.210	438	525	-	-
Substance I	−0.215	0.180	0.180	46.3	60.0	89.4	88.6
Substance II	−0.210	0.095	0.250	38.5	40.6	91.2	92.3
Substance III	−0.220	0.270	0.270	87.5	85.0	80.0	83.8
Ni-Cr alloy							
Without MCI	−0.160	0.040	0.100	233	833	-	-
Substance I	−0.180	0.230	0.145	23.2	64.8	90.0	92.2
Substance II	−0.060	0.070	0.165	-	97.8	-	88.3
Substance III	−0.205	i_lim_ **	0.105	56.7	130	75.7	84.4

* For HSS, the anodic and cathodic current densities i_a_ and i_c_ are given at E = −100 and −300 mV, respectively. For HSS, the i_a_ and i_c_ values are given at E = −80 and −300 mV, respectively; ** i_lim_—limiting current.

**Table 12:** Values of kinetic constants, the surface hydrogen coverage ($\theta_{H}$), its subsurface concentration in St3 ($C_{H}^{s}.$) and the coefficient of inhibition of hydrogen absorption by the metal ($Z_{H}^{v}.$) at a polarization potential of E = −0.3 V in 2 M HCl containing various additives.

MCIs	k_1,i_, mol/(m^2^ s)	k_r_, mol/(m^2^ s)	k, m^3^/mol	θ_H_ × 100	$C_{H}^{s}.$, mol/m^3^	$Z_{H}^{v}.$, %
Without MCI	9.73 × 10^–5^	7.50 × 10^–2^	0.35	3.4	1.00 × 10^–1^	
Substance I	1.10 × 19^–6^	6.00 × 10^–3^	2.50	1.1	3.50 × 10^–3^	96.5
Substance II	1.56 × 10^–6^	5.15 × 10^–3^	3.16	1.1	4.39 × 10^–3^	95.6
Substance III	1.50 × 10^–5^	3.00 × 10^–3^	2.60	3.3	1.50 × 10^–2^	85.0

**Table 13:** Parameters of LCS of grade 08PS based on the results of the Kelvin probe force microscopy method.

Surface Treatment	Micrograph (400 × 600 μm)	Topographic Map	Root Mean Square Roughness, s, nm	Work Function, W_s_, eV
Without treatment	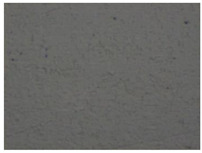	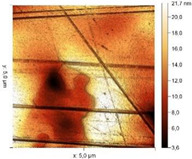	3.37	4.323
2 M HCl	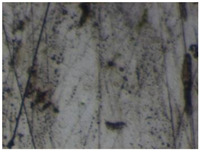	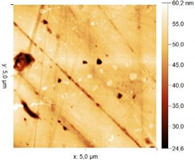	3.95	4.527
2 M HCl + Substance I	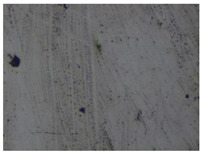	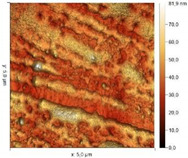	7.74	4.49
2 M HCl + Substance II	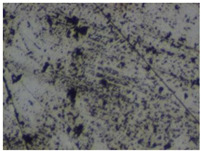	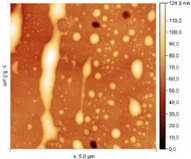	13.66	4.41
2 M HCl + Substance III	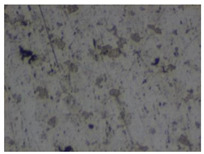	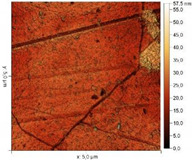	3.9	4.6

## Data Availability

The original contributions presented in this study are included in the article. Further inquiries can be directed to the corresponding author.
